# Vitamin D and Male Sexual Function: A Transversal and Longitudinal Study

**DOI:** 10.1155/2018/3720813

**Published:** 2018-01-08

**Authors:** Giacomo Tirabassi, Maurizio Sudano, Gianmaria Salvio, Melissa Cutini, Giovanna Muscogiuri, Giovanni Corona, Giancarlo Balercia

**Affiliations:** ^1^Division of Endocrinology, Department of Clinical and Molecular Sciences, Polytechnic University of Marche, Via Conca 71, Umberto I Hospital, 60126 Ancona, Italy; ^2^Diabetology and Endocrinology Unit, S. Maria della Misericordia General Hospital, Via Comandino 70, 61029 Urbino, Italy; ^3^I.O.S. & COLEMAN Srl, Acerra, 80011 Naples, Italy; ^4^Endocrinology Unit, Maggiore-Bellaria Hospital, Medical Department, Azienda USL di Bologna, Bologna, Italy

## Abstract

**Background:**

The effects of vitamin D on sexual function are very unclear. Therefore, we aimed at evaluating the possible association between vitamin D and sexual function and at assessing the influence of vitamin D administration on sexual function.

**Methods:**

We retrospectively studied 114 men by evaluating clinical, biochemical, and sexual parameters. A subsample (*n* = 41) was also studied longitudinally before and after vitamin D replacement therapy.

**Results:**

In the whole sample, after performing logistic regression models, higher levels of 25(OH) vitamin D were significantly associated with high values of total testosterone and of all the International Index of Erectile Function (IIEF) questionnaire parameters. On the other hand, higher levels of total testosterone were positively and significantly associated with high levels of erectile function and IIEF total score. After vitamin D replacement therapy, total and free testosterone increased and erectile function improved, whereas other sexual parameters did not change significantly. At logistic regression analysis, higher levels of vitamin D increase (Δ-) were significantly associated with high values of Δ-erectile function after adjustment for Δ-testosterone.

**Conclusions:**

Vitamin D is important for the wellness of male sexual function, and vitamin D administration improves sexual function.

## 1. Introduction

Erection is a complex phenomenon which implies a delicate and coordinated equilibrium among the neurological, vascular, and the tissue compartments. Many clinical factors (e.g., age, cardiovascular risk, and smoking habit) are able to impair erectile function [1].

Vitamin D is mainly involved in the regulation of calcium/phosphorus homeostasis and bone health. However, animal and human studies also suggest an involvement of vitamin D in the pathogenesis of several endocrine diseases [2–4]. In fact, vitamin D may have a role also in type 1 and type 2 diabetes mellitus, Addison's disease, Hashimoto's thyroiditis, Graves' disease, and polycystic ovary syndrome [2–4].

Some evidence also suggests a possible role for vitamin D in the regulation of sexual function. In particular, recent clinical works have suggested that low vitamin D levels may somehow be associated with the occurrence of sexual disturbances, especially erectile dysfunction [5–8], even if not all the studies agree on this point [9]. These results need confirmation because of their potential revolutionary approach to the treatment of sexual dysfunction. To the best of our knowledge, only three works have assessed the effect of vitamin D supplementation on sexual function, showing different results [8, 10, 11].

In view of these considerations, in this retrospective research, we performed a transversal and a longitudinal study, aimed at evaluating both the association between vitamin D and sexual dysfunction and the possible effect of vitamin D supplementation on sexual function.

## 2. Materials and Methods

### 2.1. Subjects

114 men [age: median 64.0 (interquartile range 60.7–68.0)] who attended our andrology unit seeking medical care for sexual dysfunctions from 2003 to the present were retrospectively evaluated.

Registration of patients who attended our andrology unit for sexual dysfunctions from 2003 to the present was retrospectively reviewed. The inclusion criteria were as follows: (1) absence of important systemic diseases, both previous and ongoing, apart from late-onset hypogonadism or type 2 diabetes mellitus, (2) availability of all the considered clinical and biochemical parameters, (3) sexual relationship since at least 1 year before the visit, (4) absence of previous investigation or treatment for sexual dysfunction, and (5) having carried out calcium metabolism assessment in the summer period (June–September) both at baseline and after treatment. The exclusion criteria were as follows: (1) ongoing pharmacological treatment for hypogonadism, (2) presence of endocrinological disorders other than late-onset hypogonadism or type 2 diabetes mellitus, (3) alcohol or drug dependence, and (4) psychiatric diseases.

Late-onset hypogonadism was defined according to the previous accepted biochemical criteria (fasting levels of serum total testosterone < 2.31 ng/mL or, in case of total testosterone between 2.31 and 3.46 ng/mL, calculated free testosterone < 65 pg/mL) together with signs and symptoms consistent with hypogonadism [12, 13].

As far as the therapy of diabetic patients is concerned, they underwent therapy with insulin (20 subjects), oral antihyperglycemic agents (38 subjects), and dietary regimen (2 subjects). Furthermore, 39 out of the diabetic patients had antidyslipidemic treatment (27 subjects treated with statins and 12 treated with omega-3 fatty acids), and 37 diabetics had antihypertensive therapy (23 subjects treated with angiotensin-converting enzyme inhibitors and 14 treated with angiotensin receptor blockers).

A subsample of 41 subjects [median age 65 (interquartile range 61–70)] was also studied longitudinally before and after vitamin D replacement therapy.

Patients were administered cholecalciferol (oral solution) at various dosages (50,000 or 100,000 IU) either weekly or every two or three weeks or monthly. In our clinic, vitamin D is supplemented when its value is less than 30 ng/mL [6]. Our study considered the time points before vitamin D therapy (time 0) and the first available visit in which normal vitamin D levels were achieved. Median follow-up duration was 9 months (total range 9–12). Clinical, biochemical, and sexual evaluations were performed at the two time points. Vitamin D-supplemented subjects during the time course of the study, although affected by hypogonadism and sexual dysfunction, were not undergoing any other pharmacological therapy for various reasons (e.g., medical decision not to begin testosterone therapy or treatment with phosphodiesterase type 5 inhibitors and patients' preference not to take phosphodiesterase type 5 inhibitors).

All the clinical and biochemical parameters were performed in the context of the clinical work-up. Proper communication of this retrospective analysis was given to our institutional review board, which approved it.

### 2.2. Clinical Parameters

The following clinical parameters were considered: age, body mass index (BMI), and presence/absence of type 2 diabetes mellitus, dyslipidemia, hypertension, and smoking habit. Of note, BMI and smoking habit were taken according to standard methods previously described [14].

### 2.3. Biochemical Parameters

The following biochemical parameters were considered: follicle-stimulating hormone (FSH), luteinizing hormone (LH), total testosterone, free testosterone, sexual hormone-binding globulin (SHBG), estradiol, parathyroid hormone (PTH), 25(OH) vitamin D, calcemia, creatinine, and albumin. Blood samples for FSH, LH, total testosterone, SHBG, estradiol, parathyroid hormone (PTH), 25(OH) vitamin D, calcemia, creatinine, and albumin were taken in the morning, and their assay was carried out as previously specified [15–18].

More specifically, as far as the total testosterone is concerned, its assay was performed using the ADVIA centaur XP immunoassay, Siemens Medical Solutions Diagnostics (minimum detectable concentration is 0.1 ng/mL, and intra- and interassay coefficients of variation were, respectively, 6.2% and 4.4% at a testosterone concentration of 0.95 ng/mL and 4.7% and 4.7% at 3.65 ng/mL).

Measurement of 25(OH) vitamin D was performed by means of a chemiluminescence assay (Liaison XL, DiaSorin, Stillwater, MN) (intra-assay coefficients of variation were 3.8% at a vitamin D concentration of 7.85 ng/mL, 2.3% at 19.6 ng/mL, and 2% at 51.9 ng/mL). Concentration of vitamin D < 20.0 ng/mL and between 20.0 to 30.0 ng/mL is adopted as “deficient” and “insufficient,” respectively, while optimal levels were defined as vitamin D > 30.0 ng/mL [6].

Free testosterone was calculated according to Vermeulen's formula (at http://www.issam.ch/freetesto.htm) [19].

### 2.4. Sexual Assessment

Sexual assessment was carried out by administering the International Index of Erectile Function-15 questionnaire (IIEF-15) [20], which considers five aspects of male sexual life: erectile function, orgasmic function, sexual desire, intercourse satisfaction (IS), and overall satisfaction. Erectile function (items 1, 2, 3, 4, 5, and 15), orgasmic function (items 9 and 10), sexual desire (items 11 and 12), intercourse satisfaction (items 6, 7, and 8), and overall satisfaction (items 13 and 14) domains were used to assess sexual function. The IIEF-15 score can indicate normal (score range 26–30), slightly impaired (score range 17–25), moderately impaired (score range 11–16), or severely impaired (score less than 11) erectile function. For orgasmic function (score range 0–10), sexual desire (score range 2–10), intercourse satisfaction (score range 0–15), and overall satisfaction (score range 2–10), the higher the score the better [20]. The total IIEF-15 score was also considered.

### 2.5. Statistical Analysis

The study is composed of two parts. One part is cross-sectional while the second is longitudinal. Each of them was defined with an exploratory sample size analysis using the software GPower (version 3.1.9.2, program written by Franz Faul, Universität Kiel, Germany). The first part has the aim of evaluating the presence of a relationship between vitamin D and erectile function. For this purpose, we based our hypothesis on a previous study [5], which reported a correlation of 0.39. Hence, the study was designed to detect the same correlation in contrast to a null hypothesis of *r* = 0 (two-sided), placing alpha level at 0.05 and setting power at 90%. This produced a sample size of 64 patients. The other part of the study used a subsample to investigate the effect of vitamin D supplementation. In this case, we started designing the study taking into account the results of Canguven et al. [10]. In particular, they assessed erectile function before supplementation and at four follow-ups. Although they found a score difference after 9 months of about 6 points, we expected as a clinically significant difference an increase of 2 points (one-tailed). Alpha level was placed at 0.05, while power was set at 90%. This produced a sample size of 29 patients.

Shapiro-Wilk's test was applied to verify the normal distribution of the continuous variables. Data are reported as mean and standard error of the mean for variables normally distributed or as median and interquartile range for variables nonnormally distributed. Statistical comparison between the two phases was made using Student's *t*-test for paired data or Wilcoxon's test depending, respectively, on normal or nonnormal data distribution. Variation of the studied parameters (Δ) was computed as the value present after vitamin D treatment minus the value present at baseline.

In the transversal part of our study, in order to analyze the independent relationship of vitamin D with testosterone levels and sexual parameters, a two-step approach was adopted. First, Spearman correlations were performed to analyze the relationships of clinical/hormonal variables with hormonal/sexual parameters. Also, statistical comparisons of the hormonal/sexual profile between the categorical clinical variables (i.e., presence/absence of type 2 diabetes mellitus, dyslipidemia, hypertension, and smoking habit) were performed (Mann–Whitney *U* test). Then, variables which were significantly correlated or categorical clinical variables which differed in the hormonal/sexual profile were entered into logistic regression models in order to assess their independent association with hormonal/sexual parameters. In order to fit with logistic regression, hormonal/sexual parameters (dependent variables) were dichotomized according to their median value. Dependent variables are categorized as 0 (low values) and 1 (high values).

In the longitudinal part of the study, the same statistical approach was adopted in order to analyze the independent relationship of the variation of vitamin D with the one of hormonal and sexual parameters. However, in this regard, only variables, which significantly varied between the two phases, were considered in the analysis.

Significance was set at *P* < 0.05. Statistical analyses were performed using SPSS 16 package (SPSS Inc., Chicago, IL, USA).

## 3. Results

### 3.1. Data Presentation

Clinical, hormonal, and sexual parameters of the total sample are shown in Table 1. After vitamin D replacement therapy, total and free testosterone increased. Erectile function improved, whereas other sexual parameters did not change significantly (Table 1). Similarly, in hypogonadic patients, total and free testosterone as well as the erectile function index significantly increased after vitamin D supplementation, whereas orgasmic function, sexual desire, intercourse satisfaction, overall satisfaction, and total IIEF score did not change significantly (data not shown).

### 3.2. Cross-Sectional Analysis in the Total Sample

Negative correlations of age with sexual parameters were found (Table 2). In addition, total testosterone and vitamin D were positively correlated with sexual function (Table 2). Most of the sexual parameters as well as testosterone levels were significantly lower in subjects with diabetes mellitus and smoking habit than in those without, whereas no difference was noticed between subjects with and without dyslipidemia and hypertension (Table 3).

After performing logistic regression models (Table 4), higher levels of 25(OH) vitamin D were significantly associated with high values of total testosterone and of all the IIEF questionnaire parameters. On the other hand, higher levels of total testosterone were positively and significantly associated with high levels of erectile function and IIEF total score.

### 3.3. Longitudinal Analysis in Supplemented Subjects

At bivariate correlation analysis, important variables, which changed significantly after vitamin D replacement therapy, were correlated among each other. Δ-vitamin D did not significantly correlate with Δ-total testosterone nor was there a significant correlation between Δ-total testosterone and Δ-erectile function (data not shown). On the other hand, positive and significant correlation was found between Δ-vitamin D and Δ-erectile function (*r* = 0.806; *P* < 0.001). Furthermore, Δ-total testosterone and Δ-erectile function did not differ significantly between subjects with and without diabetes mellitus, dyslipidemia, hypertension, and smoking habit (data not shown). At logistic regression analysis, higher levels of Δ-vitamin D were significantly associated with high values of Δ-erectile function after adjustment for Δ-testosterone [odds ratio: 2.082 (95% confidence interval 1.232–3.518); *P* = 0.006; model significance, *P* < 0.001].

## 4. Discussion

Our cross-sectional study shows that vitamin D levels are directly able to influence all sexual function parameters.

Several transversal clinical studies have evaluated and confirmed the role of vitamin D in influencing sexual dysfunction. In this regard, the widest study has been conducted by Farag and colleagues who performed a cross-sectional analysis of a nationally representative sample of 3390 US men aged ≥ 20 years. They found that 25(OH) vitamin D levels were lower in men with, than in those without, erectile dysfunction. After adjusting for comorbidities, lifestyle variables, and medication use, those with 25(OH) vitamin D deficiency had a higher prevalence of erectile dysfunction compared to those with optimal levels [7]. Interestingly, similar to our results, their findings suggest that the link of 25(OH) vitamin D with erectile dysfunction is independent of testosterone [7]. Consistently, Barassi et al. evaluated fifty patients affected by erectile dysfunction and reported that those with severe/complete erectile dysfunction were more frequent in the group with vitamin D levels < 20 ng/mL as compared with those in the group with levels > 20 ng/mL. [6]. In addition, another study found that in a large cohort of type 2 diabetic subjects, patients with lower 25(OH) vitamin D levels (<10 ng/mL) showed higher penile intima-media thickness and lower IIEF-5 score and cavernous peak systolic velocity compared to patients with 25(OH) vitamin D > 20 ng/mL. Also, 25(OH) vitamin D levels were directly correlated with IIEF-5, and, at multivariate analysis, 25(OH) vitamin D deficiency remained a predictor of erectile dysfunction independent of other confounding factors [5]. Furthermore, in a particular case series of 37 dialysis patients, the ASEX total score was found to be negatively correlated with serum 25(OH) vitamin D level, thus demonstrating that sexual dysfunction is related to low serum 25(OH) vitamin D levels [8]. Conversely, one clinical study denied this association. In fact, Bellastella and colleagues, by examining 122 male adults with type 2 diabetes (51 with associated hypogonadism and 71 with normal gonadal function), found that there was no correlation between the severity of erectile dysfunction and vitamin D levels [9].

On the other hand, longitudinal evaluation of our study clearly shows the improvement of erectile function after vitamin D replacement therapy, also demonstrating the direct influence of the vitamin D treatment. Our findings agree with the ones of a recent work, which has demonstrated the favorable effect of vitamin D supplementation on sexual function. In fact, Canguven et al., by prospectively evaluating 102 male patients with deficient serum vitamin D level (<30 ng/mL), found that vitamin D treatment induces an increase in both serum testosterone and erectile function scores [10]. However, another group, by evaluating the effect of cholecalciferol treatment in 37 dialysis patients, found no significant change in any of the studied sexual parameters [8]. Similarly, in a study by Blumberg and colleagues, in 1980, maintenance hemodialysis patients with secondary hyperparathyroidism were shown to have no improvement in the sexual parameters after 2–4 months of 1.25(OH)2 vitamin D [11].

Several mechanisms have been hypothesized to explain the effect of vitamin D on sexual function. Evidence suggests that vitamin D affects endothelial integrity. In fact, low vitamin D levels have been proved to correlate with endothelial dysfunction, and vitamin D may directly protect endothelial cells against oxidative stress [21]. One of the mechanisms involved in this process is the physiological suppression effect of vitamin D on renin, angiotensin II, and aldosterone [22]. Therefore, in vitamin D-deficient subjects, angiotensin increases and enhances nicotinamide adenine dinucleotide (phosphate) oxidase activity, resulting in the production of superoxide ions [23]. Of note, the antioxidant pathway of vitamin D is also mediated by the activation of the Mek/Erk-Sirt1 axis [24]. In addition, activated vitamin D stimulates the production of nitric oxide in endothelial cells and nitric oxide synthases, a key process to penile vascular dilation [25, 26]. Furthermore, it also plausible that vitamin D binds directly to the androgen receptor as suggested by computer (in silico) models which show that besides activating the vitamin D receptor, 1,25(OH) vitamin D also has affinity for several other nuclear receptors, such as *ɑ*/*β* thyroid receptors, glucocorticoid receptor, and androgen receptor. It can therefore be hypothesized that vitamin D at high concentrations can displace the native ligands of the receptors [10, 27]. Also, it must be remembered that vitamin D deficiency could impair erectile function by increasing cardiovascular risk, because of its correlation with obesity, diabetes mellitus, dyslipidemia, and hypertension [28].

It is also plausible that vitamin D directly affects testosterone levels. In our sample, vitamin D was found to be directly associated with testosterone levels after adjusting for confounding factors and testosterone levels increased slightly, but significantly, after vitamin D supplementation. Vitamin D receptor has been found in the testis [29], in the hypothalamus [30], and in the pituitary gland [31]. Furthermore, it has been reported that 1,25(OH)2 vitamin D is able to increase LH-induced testosterone production for both immature and mature ram Leydig cells [32]. In the same work, treatment with all doses of 1,25(OH)2 vitamin D in the absence of LH and 10 and 100 ng/mL LH in the absence of 1,25(OH)2 vitamin D increased mitochondrial dehydrogenase activity for cultured Leydig cells [32]. In addition, a trial carried out on 165 participants (54 men) observed an increase in testosterone in the vitamin D-supplemented group, whereas in the placebo group, no significant change in testosterone was found [33]. Conversely, it must also be mentioned that two different clinical studies found that vitamin D supplementation did not affect testosterone levels in men [34, 35]. However, in our sample, neither the rise of serum vitamin D correlated with testosterone increase, nor the improvement of erectile function correlated with the increase in testosterone levels. These findings make us hypothesize that the increase in testosterone levels may not be due to vitamin supplementation and that the improvement of erectile function is not due to the increase in testosterone levels.

Before concluding, we have to acknowledge that the major limitation of our work is its retrospective nature. However, it must be highlighted that it represents our 14-year experience. Furthermore, its major strength is that it includes both a transversal and a longitudinal evaluation of the sample.

In conclusion, our work shows that vitamin D is important for the wellness of male sexual function and that vitamin D administration improves sexual function. If our data will be further confirmed, vitamin D evaluation might be included in the near future in the clinical work-up protocol of male sexual dysfunction.

## Figures and Tables

**Table 1:** Clinical, hormonal, and sexual characteristics of the studied sample at baseline and after vitamin D replacement therapy.

		Baseline	Subjects who underwent vitamin D supplementation		*P* ^∗^
		Total sample (*n* = 114)	Before therapy (*n* = 41)	After therapy (*n* = 41)	
Clinical indicators	BMI	29.5 (26.1–31.3)	28 (25.7–35)	28.4 (25.4–34.9)	NS
	Hypogonadism (yes) (%)	66 (57.8)	34 (82.9)		NC
	Type 2 diabetes mellitus (yes) (%)	60 (52.6)	29 (70.7)		NC
	Dyslipidemia (yes) (%)	53 (46.4)	18 (43.9)		NC
	Hypertension (yes) (%)	51 (44.7)	16 (39)		NC
	Smoking habit (yes) (%)	68 (59.6)	34 (82.9)		NC

Biochemical indicators	FSH (IU/L)	13.6 ± 0.71	15.1 (9.3–22.9)^a^	15.5 (9.3–20.7)	NS
	LH (IU/L)	12.1 (5.7–15.6)	14.4 (11–16.7)	10.9 (8.8–16.4)	NS
	Total testosterone (ng/mL)	2.95 (2.77–4.60)	2.78 (2.27–2.95)	3.21 (2.64–3.41)	<0.001
	Free testosterone (pg/mL)	59.4 (54.2–101)	53.5 (44.3–59.3)	63.6 (53–71.3)	<0.001
	SHBG (nmol/L)	31.2 (29.3–32.6)	32.2 (28.3–34.3)	29.8 (25.3–35.7)^a^	NS
	Estradiol (pg/mL)	11.8 (7.8–29.4)	7.7 (5.5–11.6)	8.1 (6.4–11.1)	NS
	Creatinine (mg/dL)	0.53 (0.48–0.56)	0.53 (0.48–0.54)	0.54 (0.44–0.66)^a^	NS
	25(OH) vitamin D (ng/mL)	31 ± 0.59	24 ± 0.62	38.6 ± 0.89	<0.001
	PTH (pg/mL)	55.7 (49.3–60.5)	62.9 (59.9–75.5)	39.3 (29.9–49.6)^a^	<0.001
	Albumin (g/dL)	4.47 ± 0.019	4.44 ± 0.03	4.43 ± 0.047	NS
	Calcemia (mg/dL)	9.33 ± 0.020	9.30 ± 0.03	9.18 ± 0.069	NS

Functional indicators	Erectile function	18 (11–21)	10 (8–13)	11 (9–13.5)^a^	<0.001
	Orgasmic function	7 (6–8)	5 (4–6)	5 (4–6)	NS
	Sexual desire	7 (5–8)	4 (4–5.5)	5 (4–6)	NS
	Intercourse satisfaction	8 (7–13)	6 (6-7)	6 (6-7)	NS
	Overall satisfaction	4 (3–8)	3 (3-4)	3 (3-4)	NS
	IIEF total	44 (32–58)	29 (25–35.5)	30 (25.5–36)^a^	NS

Continuous variables are presented as mean and standard error of the mean if normally distributed or as median and interquartile range if not normally distributed. ^∗^Comparison of the values before and after therapy. ^a^These data are normally distributed but, in order to present the variable homogeneously, they are expressed as median and interquartile range. BMI: body mass index; FSH: follicle-stimulating hormone; LH: luteinizing hormone; SHBG: sexual hormone-binding globulin; PTH: parathyroid hormone; IIEF: International Index of Erectile Function; NC: noncomputable; NS: not significant.

**Table 2:** Bivariate correlation of general and hormonal parameters with testosterone and sexual parameters at baseline in the total sample.

	Total testosterone	Erectile function	Orgasmic function	Sexual desire	Intercourse satisfaction	Overall satisfaction	IIEF total
Age	NS	*R* = −0.199 *P* = 0.034	*R* = −0.197 *P* = 0.035	*R* = −0.243 *P* = 0.009	*R* = −0.206 *P* = 0.028	*R* = −0.213 *P* = 0.023	*R* = −0.205 *P* = 0.029
BMI	NS	NS	NS	NS	NS	NS	NS
Total testosterone	Not performed	*R* = 0.374 *P* < 0.001	*R* = 0.365 *P* < 0.001	*R* = 0.393 *P* < 0.001	*R* = 0.371 *P* < 0.001	*R* = 0.392 *P* < 0.001	*R* = 0.368 *P* < 0.001
Vitamin D	*r* = 0.463 *P* < 0.001	*R* = 0.829 *P* < 0.001	*R* = 0.816 *P* < 0.001	*R* = 0.834 *P* < 0.001	*R* = 0.814 *P* < 0.001	*R* = 0.828 *P* < 0.001	*R* = 0.823 *P* < 0.001

NS: not significant; IIEF: International Index of Erectile Function; BMI: body mass index.

**Table 3:** Comparison of hormonal and sexual function between subjects affected and not affected by type 2 diabetes mellitus, dyslipidemia, and hypertension and with or without smoking habit in the total sample.

(*n* = 114)	Diabetes mellitus		Dyslipidemia		Hypertension		Smoking habit	
	Yes	No	Yes	No	Yes	No	Yes	No
Total testosterone	2.82 (2.62–4.30)	3.96 (2.94–4.95)^∗^	2.88 (2.73–4.31)	2.97 (2.80–4.90)	2.89 (2.75–4.59)	2.97 (2.80–4.63)	2.87 (2.76–4.27)	4.12 (2.93–4.95)^∗^
Erectile function	17 (10–21)	21 (13–23)^∗^	21 (12.5–21.5)	16 (11–21)	21 (11–21)	16 (11–21)	16.5 (10–21)	21 (14–23)^∗^
Orgasmic function	7 (5–8)	8 (6–9)^∗^	8 (6–8)	7 (5.5–8)	8 (6–8)	7 (6–8)	7 (5–8)	8 (6–9)^∗^
Sexual desire	7 (4–8)	8 (6–8)	8 (5–8)	7 (4.5–8)	8 (5–8)	7 (5–8)	7 (4–8)	8 (6–8)^∗^
Intercourse satisfaction	8 (6.2–12.7)	12 (7–13)	12 (7–13)	7 (7–13)	12 (7–13)	7 (7–13)	7.5 (6–13)	12 (7–13)
Overall satisfaction	4 (3–8)	8 (4–9)^∗^	8 (4–8)	4 (3–8)	8 (3–8)	4 (3–8)	4 (3–8)	8 (4–9)^∗^
IIEF total	43 (29–57.7)	57 (36–62)^∗^	57 (34–58.5)	41 (30–58)	57 (32–58)	41 (31–58)	42 (29–58)	57 (37–62)^∗^

Categorization of the subjects in the yes/no classes has been done only according to the presence of the considered variable, independent of the coexistence of other clinical states. ^∗^*P* < 0.05, not significant if not specified. IIEF: International Index of Erectile Function.

**Table 4:** Logistic regression models assessing clinical and hormonal factors associated with high values of hormonal and sexual parameters at baseline in the total sample.

	Dependent variables							
	(*n* = 114)	Total testosterone	Erectile function	Orgasmic function	Sexual desire	Intercourse satisfaction	Overall satisfaction	IIEF total
Variables entered into models	Age	NS	NS	NS	NS	NS	NS	NS
	Total testosterone	Not included in the model	25.923 (1.033–650.351) *P* = 0.048	NS	NS	NS	NS	25.923 (1.033–650.351) *P* = 0.048
	25(OH) vitamin D	1.160 (1.066–1.261) *P* < 0.001	9.997 (2.160–46.272) *P* = 0.003	2.227 (1.558–3.182) *P* < 0.001	3.334 (1.751–6.349) *P* < 0.001	3.334 (1.751–6.349) *P* < 0.001	3.334 (1.751–6.349) *P* < 0.001	9.997 (2.160–46.272) *P* = 0.003
	Diabetes mellitus	NS	NS	NS	NS	NS	NS	NS
	Smoking habit	NS	NS	NS	NS	NS	NS	NS

	Model significance	*P* < 0.001	*P* < 0.001	*P* < 0.001	*P* < 0.001	*P* < 0.001	*P* < 0.001	*P* < 0.001

Odds ratio and 95% confidence interval are reported. NS: not significant; IIEF: International Index of Erectile Function.
